# Metallomic Approach to Mercury and Selenium in the Liver Tissue of *Psectrogaster amazonica* and *Raphiodon vulpinus* from the Brazilian Amazon

**DOI:** 10.3390/ijms252211946

**Published:** 2024-11-07

**Authors:** Izabela Bataglioli, José Vieira, Joyce da Siva, Luane Andrade, Victor Faria, Rebeca Corcoba, Ronaldo de Almeida, Luiz Zara, Marília Buzalaf, Jiri Adamec, Pedro Padilha

**Affiliations:** 1 School of Veterinary Medicine and Animal Science, São Paulo State University (UNESP), Botucatu 18618-681, SP, Brazil; izabela.bataglioli@unesp.br (I.B.); joyce.silva@unesp.br (J.d.S.); luane.andrade@unesp.br (L.A.); rebeca.orrantia@unesp.br (R.C.); 2 Institute of Biosciences, São Paulo State University (UNESP), Botucatu 18618-693, SP, Brazil; cavalcante.vieira@unesp.br (J.V.); v.faria@unesp.br (V.F.); 3 Wolfgang C. Pfeiffer Environmental Biogeochemistry Laboratory, Federal University of Rondônia, Porto Velho 76801-974, RO, Brazil; ronaldoalmeida@unir.br; 4 College of Planaltina, University of Brasília (UNB), Planaltina 70842-970, DF, Brazil; 5 Department of Biochemistry, Bauru School of Dentistry, University of São Paulo (USP), Bauru 17012-901, SP, Brazil; mbuzalaf@fob.usp.br; 6 School of Medicine, Louisiana State University Health Sciences Center (LSUHSC), New Orleans, LA 70112, USA; jadame@lsuhsc.edu

**Keywords:** metal binding proteins, Se/Hg-associated protein, metalloproteomics, Hg-induced oxidative stress, Hg and Se in Amazon fish

## Abstract

This paper reports the results of a mercury (Hg) and selenium (Se) metallomic study in the liver tissues of *Psectrogaster amazonica* and *Raphiodon vulpinus* from the Brazilian Amazon. Two-dimensional electrophoresis, graphite furnace atomic absorption spectrometry, and liquid chromatography-tandem mass spectrometry were performed. Hg and Se determinations allowed the calculation of Hg:Se and Se:Hg molar ratio and Se values for health benefits (Se HBVs). The Se:Hg values were >1 for both fish species, whereas the Se HBVs were >5 for *P. amazonica* and >10 for *R. vulpinus*, indicating that both possess Se reserves to control Hg toxicity. The metallomic data allowed the identification of 11 Hg/Se-associated protein spots in the two fish species, with concentrations in the range of 9.70 ± 0.14 and 28.44 ± 0.31 mg kg^−1^ of Hg and 16.15 ± 0.21 and 43.12 ± 0.51 mg kg^−1^ of Se. Five metal binding proteins (*MBP*) in the Hg/Se-associated protein spots in the liver proteome of *P. amazonica* and eight in *R. vulpinus* were identified, indicating the possible formation of Hg/Se complexes on the *MBP* structures. The activities analysis of catalase, superoxide dismutase, GPx enzymes, and lipoperoxide concentrations demonstrated that Hg-induced oxidative stress did not occur, possibly because both fish species possess Se reserves necessary to inhibit the Hg’s deleterious effects.

## 1. Introduction

Mercury (Hg) is highly toxic and accumulates throughout the food chain, posing a risk to top consumers such as humans [1]. In the Amazon, both Au mining and natural sources contribute to Hg contamination [2]. Fish consumption is the main source of contamination in traditional populations of the Amazon region, especially those who live in remote areas and depend on fish as their main source of dietary protein [3,4]. Studies have shown a positive correlation between fish consumption and Hg concentrations in the hair and blood of the traditional populations in the Amazon, thereby indicating an increased risk to these populations [5,6]. Furthermore, Hg affects fish health and reproduction, leading to a reduction in fish stocks, and negatively affects the riverine communities [7]. Owing to its antioxidant properties, selenium (Se) has been suggested as a possible contributor to the apparent tolerance of Hg in organisms. The action of Se in the detoxification of organisms in relation to Hg has been extensively reported, indicating that Se can play a crucial role in protecting against the harmful effects of Hg [8,9,10,11]. Specifically, in relation to fish, the literature reports that Se can reduce Hg species toxicity in these organisms because of its greater affinity to Hg compared to sulfur [12,13]. Organisms with a Se:Hg ratio >1 show a reduction in Hg toxic effects [12,14]. Thus, the high binding affinity of Se to Hg may trigger the protective potential of Se, reducing Hg bioavailability and possibly its toxicity to organisms [13].

Although several studies have reported the toxic effects of Hg species on humans and animals, studies that discuss the possible mechanisms of the antagonistic effects of Se on the deleterious effects of Hg are still scarce [15]. Metallomics is a relatively new line of scientific investigation, as it encompasses experimental analytical chemistry strategies along with biochemical strategies and molecular biology, and allows the identification of proteins associated with Hg species in the proteome of different organisms, such as fish [16,17]. In this context, metallomics can generate important information regarding the mechanisms related to the protective effect of Se against Hg toxicity because it allows for the identification of proteins associated with Hg and Se with differences in relative abundance in the studied proteome [18,19].

Thus, we report the results obtained from Hg and Se metallomic studies in the liver tissue of *P. amazonica* (non-predator species) and *R. vulpinus* (predator species) Amazonian fish. Both fish species were collected from the Madeira River, Rondonia State, Brazil, a river affected by Hg in recent decades owing to gold mining activities [16].

## 2. Results and Discussion

### 2.1. Mercury and Selenium Determination

Hg_total_ and Se_total_ were determined from eight liver tissue and protein pellet samples from *P. amazonica* and *R. vulpinus*. In this case, determinations were performed on livers extracted from eight individuals, from which three samples of approximately 100 mg of each liver tissue were separated. After mineralization of the samples, they were quantified using GFAAS according to the procedure described in Section 2.2. For the protein pellets, extraction and precipitation of the protein fraction were carried out in four pools of liver tissue samples. In this case, a pool with samples from individuals with lower concentrations of Hg_total_ and Se_total_ and a pool with samples from individuals with higher concentrations of Hg_total_ and Se_total_, for the two species of fish, resulted in a total of four pools. The results obtained are summarized in Table 1, Table 2 and Table 3.

The results summarized in Table 1 and Table 2 allow us to separate two groups of individuals for the two fish species depending on their concentrations of Hg_total_ and Se_total_. In this case, one group had lower concentrations of Hg_total_ and Se_total_, whereas the other had higher concentrations of Hg_total_ and Se_total_. Thus, for *P. amazonica*, the group of individuals 1, 3, 6, 7, 8, and 10 presented lower mean concentrations of Hg_total_ and Se_total_, in this case, 85.80 ± 2.602 µg kg^−1^ and 127.6 ± 3.184 µg kg^−1^, respectively. This group was denoted as Pa-HgSe 1 (<Hg_total_ and Se_total_ concentrations). The group of individuals 2, 4, 5, and 9 of *P. amazonica* presented average concentrations of 123.1 ± 1.936 µg kg^−1^ and 150.7 ± 2.800 µg kg^−1^ of Hg_total_ and Se_total_, respectively; these concentrations are higher than those of the Pa-HgSe 1 group. This group is denoted as Pa-HgSe 2 (>Hg_total_ and Se_total_ concentrations). In relation to *R. vulpinus*, the results of the average concentrations of Hg_total_ and Se_total_ also made it possible to assemble two distinct groups. The first was a group of individuals 1, 3, 4, 7, and 10, denominated Rv-HgSe 1, with <Hg_total_ and Se_total_ average concentrations (Hg_total_ and Se_total_ average concentrations = 145.4 ± 7.394 µg kg^−1^ and 242.7 ± 12.20 µg kg^−1^, respectively). A second group, denominated Rv-HgSe 2, was made up of individuals 2, 5, 6, 8, and 9 of *R. vulpinus*, group that presented > Hg_total_ and Se_total_ average concentrations (Hg_total_ and Se_total_ average concentrations = 571.7 ± 46.73 µg kg^−1^ and 692.7 ± 56.40 µg kg^−1^, respectively). The Hg_total_ and Se_total_ concentrations allowed the calculation of the molar ratios of Hg:Se and Se:Hg for the four groups of individuals of *P. amazonica* and *R. vulpinus*. The average values of the molar ratio Hg:Se and Se:Hg are: Pa-HgSe 1 group (*P. amazonica* with <Hg_total_ and Se_total_ concentrations)—Hg:Se = 0.27, Se:Hg = 3.8; Pa-HgSe 2 group (*P. amazonica* with >Hg_total_ and Se_total_ concentrations)—Hg:Se = 0.31, Se:Hg = 3.1; Rv-HgSe 1 group (*R. vulpinus* with <Hg_total_ and Se_total_ concentrations)—Hg:Se = 0.24, Se:Hg = 4.2; Rv-HgSe 2 group (*R. vulpinus* with >Hg_total_ and Se_total_ concentrations)—Hg:Se = 0.33, Se:Hg = 3.0. From the average molar ratios of Hg:Se and Se:Hg, Se values for health benefits (Se HBV) were calculated using Equation (1) [13] as follows:

Se HBV = (molar ratio Se:Hg × Se (μmol kg^−1^)) − (molar ratio Hg:Se × Se (μmol kg^−1^)) (1)

The calculated results for Se HBV were as follows: *Pa*-HgSe 1 group (*P. amazonica* with <Hg_total_ and Se_total_ concentration)—Se HBV = 6.04; *Pa*-HgSe 2 group (*P. amazonica* with Hg_total_ and Se_total_ concentration)—Se HBV = 5.73; *Rv*-HgSe 1 group (R. *vulpinus* with <Hg_total_ and Se_total_ concentration)—Se HBV = 12.73; and *Rv*-HgSe 2 group (*R. vulpinus* with <Hg_total_ and Se_total_ concentration)—Se HBV = 25.4. The calculated values for the molar ratio Se:Hg were >1 for the two species of fish, >5 for Se HBV for the two groups of *P. amazonica*, and >10 for the two groups of *R. vulpinus*. These results show that the non-carnivorous *P. amazonica* and carnivorous *R. vulpinus* have Se reserves (soft bases) in the liver tissue to form bonds with Hg species (soft acids), forming Se-Hg complexes [20,21]. Based on the results obtained for Hg_total_ and Se_total_ determinations in the liver tissue of both *P. amazonica* and *R. vulpinus*, the protein fraction was extracted from a pool of liver tissue from the *Pa*-HgSe 1, *Pa*-HgSe 2, *Rv*-HgSe 1, and *Rv*-HgSe 2 groups, and subsequently Hg_total_ and Se_total_ were determined in the respective protein pellets by GFAAS. These results are summarized in Table 3.

The Hg_total_ and Se_total_ concentrations determined in the protein pellets of *Pa*-HgSe 1, *Pa*-HgSe 2, *Rv*-HgSe 1, and *Rv*-HgSe 2 groups agreed with the results obtained in the liver tissue samples. In this case, the Hg_total_ and Se_total_ concentrations were higher in the *Pa*-HgSe 2 and *Rv*-HgSe 2 groups compared than in the *Pa*-HgSe 1 and *Rv*-HgSe 1 groups. Protein pellets obtained from the protein fractions extracted from the pools of the Pa-HgSe 2 and Rv-HgSe 2 groups (groups with >C_Hgtotal_ and C_Setotal_) of the liver proteome of the *P. amazonica* and *R. vulpinus* were subjected to 2D PAGE. The results of Hg_total_ and Se_total_ determinations in the certified reference material DOLT-4 (fish liver) showed a recovery percentage of approximately 98% and a relative standard deviation of <2, which proves the robustness of the method used for Hg_total_ and Se_total_ determinations [17,18].

### 2.2. Liver Proteome Fractionation Using 2D PAGE

**Figure 1:**
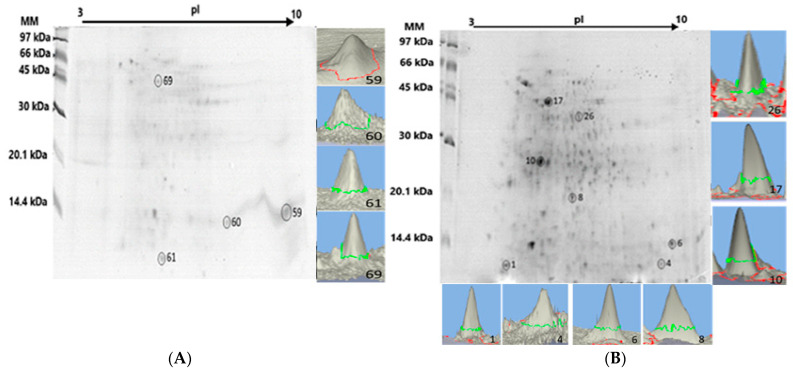
2D PAGE gel of the Pa-HgSe 2 and Rv-HgSe 2 groups obtained from the liver proteome fractionation of *P. amazonica* (**A**) and *R. vulpinus* (**B**). The spots highlighted with a circle and in three dimensions, the GFAAS determinations indicated the presence of Hg and/or Se.

The liver proteome fractionation using 2D PAGE was performed on protein pellets extracted from the liver tissue pool of the *Pa*-HgSe 2 and *Rv*-HgSe 2 groups (groups chosen for presenting > C_Hgtotal_ and C_S_e_total_), with the aim of mapping the presence of Hg and Se in the protein spots [18]. Figure 1 shows an example of a gel obtained from triplicate analysis using 2D PAGE of the liver tissue samples of the *Pa*-HgSe 2 and *Rv*-HgSe 2 groups. Hg and Se mapping was performed after acid mineralization of the protein spots and analysis using GFAAS. Proteins associated with Hg and Se were characterized using LC-MS/MS. The results are summarized in Table 4.

The GFAAS determinations (Figure 1A,B, Table 4) indicated four protein spots associated with Hg and Se in the liver proteome of the Pa-HgSe group 2 of *P. amazonica*, with concentrations ranging from 9.70 to 19.72 mg kg^−1^ and from 16.15 to 35.44 mg kg^−1^ for Hg and Se, respectively. In the liver proteome of the *R. vulpinus* (Rv-HgSe group 2), seven protein spots associated with Hg and Se were identified, with concentrations ranging from 14.35 to 28.44 mg kg^−1^ and 26.82 to 43.12 mg kg^−1^, respectively, for Hg and Se.

The results presented in Table 4 show that, in all protein spots associated with Hg, the GFAAS results also indicated the presence of Se. The Hg concentrations were over 50% lower than the Se concentrations. This pattern was similar to the results obtained for the tissues and protein pellets of the liver samples from the two fish species studied. The results of this study corroborate those in the literature, which reported a positive correlation between Hg and Se concentrations in fish from the Tapajos River region, fish from the Brazilian Amazon, and marine fish [8,14]. The positive correlation between Hg and Se can be explained by the formation of [Hg^2+^]–[Se^2−^] and [CH_3_Hg^+^]_2_–[Se^2−^], which can neutralize the deleterious effects of Hg species [13]. The data presented in Table 4 provide new information for this discussion; in this case, the identification of Hg and Se associated with the same protein spots. Studies have identified Hg-associated protein spots in the liver tissues of rats and fish [17,20]; however, the presence of Se has not been investigated in these studies. Thus, based on the data in Table 4, it can be suggested that Hg and Se may be linked to the structures of proteins and/or enzymes, possibly to the thiol group of a cysteine and/or thioether of methionine, forming inert complexes linked to *metal binding proteins* (*MBP*) of the type *MBP*-R-[Hg-Se] or *MBP*-R_2_-S-[Hg-Se]. In this case, the R-S-H group (soft base) represents the thiol group of cysteine, and the R_2_-S-H group (also a soft base) represents the thioether group of methionine in the protein structure. The [Hg-Se] species act as soft acids that bind to thiol and/or thioether groups [21]. Therefore, based on the data obtained in this study, it can be inferred that the Se_total_ concentrations determined in the liver tissue samples from *P. amazonica* and *R. vulpinus* could mitigate the toxic effects of Hg species.

### 2.3. Characterization of Hg/Se-Associated Protein Spots Using LC-MS/MS

LC-MS/MS analyses were used to identify five proteins and/or enzymes in four Hg/Se-associated protein spots and eight Hg/Se-associated protein spots in the liver proteome of *P. amazonica* and *R. vulpinus*, respectively. The results of the characterization of Hg/Se-associated protein spots are summarized in Table 4 too.

Hemoglobin subunits alpha and beta (HbA and HbB) were identified in spots 59 and 60 of the liver tissue of *P. amazonica*, which presented Hg and Se concentrations ranging from 9 to 17 mg kg^−1^ and 16 to 33 mg kg^−1^, respectively. The isoforms of hemoglobin subunits participate in oxygen transport from the gills to various peripheral tissues in fish [18,20]. A recent study by Almeida et al. (2024) [18] characterized HbA and HbB as Hg-binding proteins in the renal tissues of rats exposed to mercuric chloride at concentrations above 100 mg k^−1^ of Hg_total_. In this study, the authors highlighted that cysteine residues (amino acids that present thiol groups as soft bases) in the structure of Hb isoforms allow the binding of Hg species (soft acids). The results obtained by Almeida et al. (2024) [18] corroborated those obtained by Gibson et al. (2017) [22], who identified the binding of Hg^2+^ to the hemoglobin structure in rabbit red blood cell lysates using SEC-ICP-AES. Notably, in both studies the authors did not analyze the presence of Se in the tissues studied. Based on the results of these two studies, one hypothesis for the mechanism involved in the binding of Hg and Se in the structure of HbA and HbB is the formation of the R-S-[Se-Hg] complex, where R-S-H represents the thiol group of methionine residues present in the structure of hemoglobin isoforms, as previously discussed (Section 3.2).

Spots 60 and 61 of the liver tissue of *P. amazonica*, the fatty acid-binding protein_liver (FABP1), and, in spot 61, the isoform fatty acid-binding protein 10-A_liver basic (FABP 10a) were identified. Spots 60 and 61 presented concentrations of 17.24 ± 0.23 and 19.72 ± 0.25 mg kg^−1^ for Hg, 33.41 ± 0.39 mg kg^−1^, and 35.44 ± 0.43 mg kg^−1^ for Se, respectively. One of the main functions of fatty acid-binding proteins (FABPs_liver) is to act as antioxidants against the increase in ROS production caused by xenobiotics in the liver [16,23]. Notably, basic FABPs_L, such as FABP 10a), are found only (so far) in non-mammalian vertebrates [24]. There are numerous isoforms of FABPs; however, all isoforms contain at least one cysteine residue, several methionine residues, and amino acids that contain thiol and thioether groups (soft bases), which may explain the affinity of FABPs for Hg species (soft acids) [21,23]. Metalloproteomic studies of Hg in the liver tissue of fish from the Brazilian Amazon [7,16,25] have reported the identification of FABPs in the liver associated with Hg species; however, the authors did not discuss the effects of Hg species on the functions of FABPS-liver. In this study, FABP1 and FABP 10a isoforms were identified as Hg- and Se-*binding proteins*, respectively. In this case, it can be suggested that the presence of Se together with Hg possibly forms a Se/Hg-FAPAs_liver-type *binding protein*; thus, it is reasonable to infer that the presence of Se in the Se/Hg-FAPAs_liver complex could eliminate the possible negative effects of Hg on the functions of FAPAs_liver.

Actin_cytoplasmic 1 (ACTB1) and Actin_alpha skeletal muscle (ACTA1) were identified in spots 69 and 10 of the liver tissue of *P. amazonica* and *R. vulpinus*, respectively. In spots 69 and 10, GFAAS determinations indicated concentrations in the ranges of 14–28 and 25–43 mg kg^−1^ for Hg and Se, respectively. Actin can present different isoforms depending on its monomeric or polymeric forms, and all actin isoforms participate in essential functions in the cytoplasmic cytoskeleton. In the nucleus, actin participates in the regulation of gene transcription, motility, and DNA repair [24]. A metalloproteomic study by Vieira et al. (2023) [17] on the muscle tissue of *Cichla* sp. from the Brazilian Amazon identified Hg-associated protein spots, in which actin alpha skeletal muscle and actin cytoplasmic 1 and 2 were identified. The authors highlighted that the binding of Hg species to the structure of actin isoforms could trigger changes in the structure of the cytoskeleton, increase intracellular calcium levels, and increase autophagy. Actin isoforms (such as ACTB1 and ACTA1) present cysteine and methionine residues in their structures because they present thiol and thioether groups (soft bases), and can form stable complexes with Hg species (soft acids), as discussed above. In spots 69 and 10, in which ACTB1 and ACTA1 were identified, respectively, the GFAAS analysis results indicated the presence of Se and Hg. Thus, it can be suggested that the possible binding of [Se-Hg] complexes in the structure of ACTB1 and ACTA1 forms *metal binding proteins* (*MBPs*), such as Se/Hg-ACTB1 and Se/Hg-ACTA1, and that the presence of Se in *MBPs* could neutralize the negative effects of the Hg species on the functions that ACTB1 and ACTA1 perform in the cell cytoplasm.

The enzyme superoxide dismutase [Cu-Zn] (SOD1) was identified in spot 1 of the liver tissue of *R. vulpinus*, a spot that presented 22.11 ± 0.31 mg kg^−1^ of Hg and 34.82 ± 0.46 mg kg^−1^ of Se. SOD1 mainly inhibits free radicals produced in cells (free radicals are toxic to biological systems), such as in the process of dismutation of superoxide radicals into molecular oxygen and hydrogen peroxide [16,26]. A metalloproteomic study by Bataglioli et al. (2019) [7] using liver tissue from *Arapaima gigas* and Bittarello et al. (2020) [16] using kidney tissue from *Plagioscion squamosissimus*, a predatory species from the Brazilian Amazon, identified SOD1 in Hg-associated protein spots. Notably, the total Hg concentration determined in the protein spot of the liver tissue of *Arapaima gigas* [7] was 29.70 ± 0.53 mg kg^−1^, which can be considered close to the value determined in the present study (22.11 ± 0.31 mg kg^−1^). Both studies [7,16] highlight that the binding of Hg species to the SOD1 structure can cause structural changes, possibly owing to the covalent bond with Hg and the possible release of Cu and Zn responsible for the biological activity of SOD1, which compromises the enzyme activity. The coordination of Zn and Cu in the SOD1 structure occurs by binding to six histidine residues and one aspartate residue, forming a binary complex of Zn and Cu [26]. Considering that histidine and aspartate have amine groups, R-NH_2_, an intermediate base that coordinates strongly with intermediate acids such as Zn and Cu, and carboxylic acid (R-COOH), a hard base [21], it is unlikely that Hg species (soft acid) could displace Zn and Cu from the structure of SOD 1. However, SOD 1 also contains cysteine and methionine residues in its structure, and the amino acids have thiol and thioether groups (soft bases) that strongly coordinate with Hg species (soft acids). In spot 1 of the liver tissue of *R. vulpinus*, in which SOD 1 was identified, GFAAS analysis indicated the presence of Hg and Se, a result that has not yet been reported in the literature. Thus, it can be inferred that Se possibly forms a Se/Hg-SOD 1 type complex with thiol and thioether groups present in the enzyme structure. Thus, the formation of the *metal binding protein* Se/Hg-SOD1 could inhibit the negative action of Hg species on the biological activity of SOD1. This inference corroborates the results of the determination of SOD activity in liver tissue samples of *R. vulpinus* (results that will be presented in Section 3.4), results that did not present variation between the *Rv*-HgSe 1 group (*R. vulpinus* group with <concentration of Hg_total_ and Se_total_) and the *Rv*-HgSe 2 group (*R. vulpinus* group with >concentration of Hg_total_ and Se_total_).

The enzyme triosephosphate B isomerase (TPI 1B) was identified in spots 4 and 6 of the liver proteome of *R. vulpinus*, at 24.20 ± 0.26 and 23.12 ± 0.33 mg kg^−1^ for Hg and 39.20 ± 0.51 and 37.60 ± 0.49 mg kg^−1^ for Se, respectively. TPI 1B plays a fundamental role in homeostasis because it catalyzes the interconversion of dihydroxyacetone phosphate and D-glyceraldehyde-3-phosphate in the glycolytic and glycogenic pathways and indirectly participates in carbohydrate biosynthesis [17,24]. TBIs have several isoforms that are not associated with the coordination of metal species [24]. However, studies report the identification of Hg-*binding proteins* in spots of the liver proteome of *Arapaima giga* [7], *Cichla sp*, and *Brachyplathystoma filamentosum* [17] species from the Brazilian Amazon. Both studies highlighted that the coordination of Hg species in the structure of TBIs can be explained by the presence of cysteine and methionine residues (soft bases) in the FASTA sequence of this enzyme, forming a *metal binding protein*, in this case, Hg-TBs. As TBIs have several specific functions, the association of this enzyme with mercurial species can cause irreversible damage to organisms [17]. Considering that, in the present study, TBI 1 B was identified in spots 4 and 6 of the liver proteome of *R. vulpinus*, which are associated with Hg and Se, it can be concluded, based on previous reports, that the possible formation of the *metal binding protein* Se/Hg-TBI 1 B could nullify the negative effects of Hg species on the different functions that TBI 1 B performs in organisms.

Keratin_type II cytoskeletal 8 (KRT8) was identified in spot 26 of the liver tissue of *R. vulpinus* with a concentration of 14.35 ± 0.21 mg kg^−1^ of Hg and 26.82 ± 0.31 mg kg^−1^ of Se. KRT8, together with KRT19, helps link the contractile apparatus to dystrophin in the costameres of the striated muscles. KRT8 is present in the FASTA sequence of several methionine residues, an amino acid which contains thioether groups. Thioether groups are soft bases with a high affinity for soft acids such as Hg species, which may explain the association between Hg and KRT8 [21,24]. Vieira et al. (2015, 2023) [17,27] used metalloproteomic strategies to identify the Hg associated with KRT8 and estimated the ratio of two Hg atoms per KRT8 molecule, which corroborated the data of the present study. Thus, it can be suggested that the *metal binding protein* Se/Hg-KTR8, due to the coordination of Se, could suppress the negative interference of Hg in the biological functions of HRT8.

The enzymes 4-hydroxy-2-oxoglutarate aldolase_mitochondrial (HOGA), ATP synthase subunit beta_mitochondrial (ATP5F1B), and the protein Intermediate filament protein ON3 (IFP ON3) were identified in spots 8 (C_Hg_ = 21.43 ± 0.28 mg kg^−1^; C_Se_ = 33.73 ± 0.43 mg kg^−1^), 17 (C_Hg_ = 17.32 ± 0.23 mg kg^−1^; C_Se_ = 31.25 ± 0.41 mg kg^−1^), and 26 (C_Hg_ = 14.35 ± 0.21 mg kg^−1^; C_Se_ = 26.82 ± 0.31 mg kg^−1^), respectively. The main function of the HOGA enzyme is to catalyze the final step of the hydroxyproline metabolic pathway in mitochondria [24,28]. ATP5F1B is responsible for the production of ATP from ADP in the presence of a proton gradient across the membrane, which is generated by the electron transport complexes of the respiratory chain [24]. IFP ON3 is a part of a set of cytoplasmic and nuclear cytoskeletal proteins. Intermediate filament proteins adapt to post-translational modifications, adapting and reflecting different functional states of a given cell [29]. To date, metalloproteomic studies on HOGA, ATP5F1B, or IFP ON3 in the presence of Hg and Se have not been reported. Thus, the data reported in this study are pioneering. HOGA, ATP5F1B, and IFP ON3 contain cysteine and/or methionine residues in their FASTA sequences [24]. As previously discussed, cysteine and methionine have a high affinity for Hg and Se. Thus, it can be inferred that Se and Hg can be coordinated to the thiol and/or thioether groups of HOGA, ATP5F1B, and/or IFP ON3, forming Se/Hg-HOGA, Se/Hg-ATP5F1B, and Se/Hg-IFP ON3 complexes. Therefore, the lack of studies reporting the interactions of HOGA, ATP5F1B, and IFP ON3 with Hg and Se reinforces the need for specific studies on these interactions, considering the new insights reported in this study.

### 2.4. Oxidative Stress Parameters

**Figure 2:**
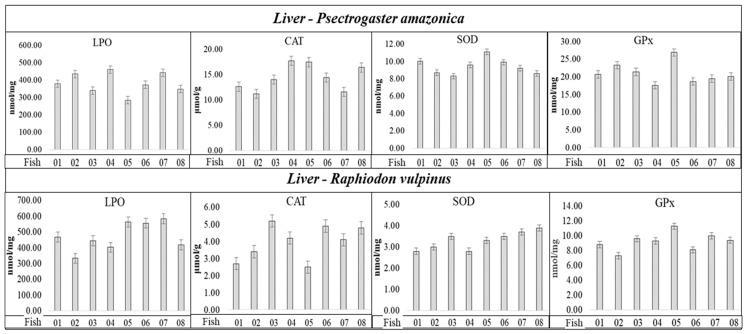
Activities of SOD, CAT, and GPx enzymes, and LPO concentration determined in liver tissue of individuals of the *P. amazonica* and *R. vulpinus*.

The antioxidant enzymes, such as CAT, GPx, and SOD, are the first line of defense against the increase in reactive oxygen species in organisms, and their levels were analyzed with the aim of investigating whether Hg concentrations in *P. amazonica* and *R. vulpinus* could trigger a state of oxidative stress. The results are summarized in Figure 2 and Table 5.

Analyzing the results in Figure 2, the values related to the SOD, CAT, and GPx activities, and LPO concentration, apparently do not present a correlation with Hgtotal and total concentrations, considering the individuals with the lowest and highest Hg_total_ and Se_total_ concentrations (Table 1 and Table 2) in the *P. amazonica* and *R. vulpinus* species. It was possible to observe among the individuals that presented the lowest and/or highest Hg_total_ and Se_total_ concentrations (Table 1 and Table 2) very close maximum and minimum values of CAT, SOD, and GPx activities and LPO concentrations (Figure 2 and Table S1, Supplementary Materials). Spearman’s correlation coefficient (Sp.CC) values, calculated by correlating the lowest concentrations of Hg_total_ and Se_total_ with the activities of CAT, SOD, and GPx, and with LPO concentration (Table 5), followed the same trend, presenting six significant values (*p* < 0.05) with CCSp values (+) in the range of 0.61 to 0.84 and eight values (−), in the range of −060 to −0.88. Most Sp.CC (+) and/or (−) values were not significant (*p* ≥ 0.05).

Regarding the LPO concentrations for the two fish species, the groups with <Hg_total_ and Se_total_ concentrations presented significant Sp.CC (+); however, the groups with >Hg_total_ and Se_total_ concentrations presented non-significant Sp.CC. Regarding SOD in *P. amazonica*, Sp.CC values (+) and significance for groups with <Hg_total_ and Se_total_ concentrations were observed, and Sp.CC values (−) and significance for groups with >Hg_total_ and Se_total_ concentrations. Regarding SOD in *R. vulpinus*, the Sp.CC values were not significant in either group. The Sp.CC values for CAT were also not significant in any of the groups of *P. amazonica* and *R. vulpinus*. As for GPx, in relation to the *P. amazonica*, Sp.CC values (−) were observed and significant for the groups with >and < Hg_total_ and Se_total_ concentrations, whereas for the *R. vulpinus*, the Sp.CC values were not significant for the groups with <Hg_total_ and Se_total_ concentrations; however, in relation to the groups with higher concentrations, the Sp.CC values were significant and presented a negative correlation. Bittarello et al. (2020) [16], working with *Plagioscion squamosissimus* (carnivorous) and *Colossoma macropomum* (omnivorous) from the Brazilian Amazon, found a trend of greater enzymatic activity and LPO concentration in the liver tissue of *Plagioscion squamosissimus*, which presented a higher Hg concentration. The calculated Sp.CC values demonstrate only the occurrence of a negative correlation between GPx activity and Hg_total_ concentration (Sp.CC = −0.7040, C_Hgtotal_ = 279 ± 4.41 µg kg^−1^), which partially corroborates the data reported in this study. The authors inferred that the inhibition of GPx by Hg could be related to the Hg–selenol interaction. However, in that study, Se determination was not performed in the liver tissue samples of the fish, which would provide greater support for the results obtained. In the present study, Se determination allowed the calculation of HBV Se values, which were five times higher for *P. amazonica* (non-predatory species, which presented the lowest Hg concentration) and ten-fold higher for *R. vulpinus* (predatory species, which presented the highest Hg concentration). Thus, the results reported here provide new insights into the influence of Se on the capacity of Hg to generate oxidative species, and the possible triggering of oxidative stress. It should be highlighted that SOD was identified in spot 1 of the liver tissue of *R. vulpinus*, a spot that showed 22.11 ± 0.31 mg kg^−1^ of Hg_total_ and 34.82 ± 0.46 mg kg^−1^ of Se_total_ (Table 5). However, Table 5 shows that there is no significant correlation between SOD activity and Se_total_ and Hg_total_ concentrations for *R. vulpinus* specie.

## 3. Material and Methods

### 3.1. Samples Processing and Collection 

Liver tissue samples from 10 fish each of *P. amazonica* (branquinha) and *R. vulpinus* (cachorra) were collected and preserved in liquid nitrogen in the Jirau Hydroelectric Plant reservoir in the Rondonia state of Brazil. The collection points were: (a) (S 09°47′18.3″; W 065°31′12.4″), (b) S 09° 41′0.06″; WE 064° 58′43.9″, and (c) S 09°16′12.8″; W 064°41′14″), located on the Madeira River [16,20]. The fish were collected considering the size and weight of the species after approval by Chico Mendes Institute for Biodiversity Conservation and Authorization and Information System on Biodiversity (ICMBio; Ref. SISBIO 43890-1) and the Committee on Ethics in the Use of Animals—UNESP (CEUA-UNESP), protocol number n^o^ 0316/2023. The fish were captured with meshes by a team of biologists from Empresa ESBR (Empresa Energia Sustentável do Brasil), euthanized by myelotomy to perform biometry, and tissue samples were collected [7,16].

### 3.2. Hg and Se Determinations 

Hg and total Se (Hg_total_ and Se_total_) in the liver tissue samples, pellets, and protein bands were determined using graphite furnace atomic absorption spectrometry (GFAAS), according to the procedure described by Silva et al. (2007) and Vieira et al. (2023) [17,30]. The samples were mineralized using a mixture of 70% (m/m) nitric acid (J Backer) with a 0.05 mol L^−1^ potassium permanganate (Merck, Rahway, NJ, USA), solution and heating at 120 °C in a digester block until a slightly yellowish transparent extract was obtained. After the mineralization process, the extracts were transferred to 5.00 mL volumetric flasks, and the volumes were adjusted with ultrapure water (18.2 MΩ cm). In the analysis stage, to maintain the Hg and Se thermal stabilization at the atomization temperature in the graphite furnace, a combination of 1000 mg L^−1^ sodium tungstate solution (Merck, Rahway, NJ, USA) was used as a permanent chemical modifier and 100 mg L^−1^ zirconium chloride (Sigma-Aldrich, San Louis, MO, USA) as a chemical modifier, and co-injected with the sample and/or standard solution into the graphite tube of the spectrometer [18,20]. The measurements were performed using a SHIMADZU AA-6800 atomic absorption spectrometer (Osaka, Japan) equipped with a background corrector with a self-reverse system and a deuterium lamp, a pyrolytic graphite tube with an integrated platform, and an AS6100 automatic sampler. All Hg_total_ and Se_total_ determinations were validated by analyzing the National Research Council of Canada (NRC) DOLT 4 (fish liver) certified reference material. Based on the data obtained from the Hg_total_ and Se_total_ determinations in the liver tissue samples, the ratios of Hg:Se and Se:Hg, and Se values for health benefits (Se HBVs) were calculated [13].

### 3.3. Liver Proteome Fractionation Using 2D PAGE

Liver proteome fractionation of *P. amazonica* and *R. vulpinus* was performed using 2D PAGE. For this purpose, sample pools were assembled based on the Hg and Se concentrations per individual for both fish species. Thus, based on the Hg_total_ and Se_total_ concentrations per individual, the two groups of pools were elaborated in increasing order of concentration (group 1: lowest Hg and Se concentrations and group 2: highest Hg and Se concentrations) for the two fish species. Protein fraction extraction using the liver tissue samples was carried out with Tris-HCl buffer pH 8.80 under denaturing conditions according to the procedure described by Bataglioli et al. (2019) [7], using an OMNI/analytic cell disruptor (Kennesaw, GA, USA). To remove concomitants of the protein extracts that could interfere with the proteome fractionation process, and also more abundant proteins of no interest in the metalloproteomic study (such as albumin), the fractional precipitation strategy was used [7,27]. Initially, an ethanol–chloroform (Merck, Rahway, NJ, USA) mixture (12.5:1 *v*/*v*) was used to precipitate proteins with higher molecular mass greater than 90 kDa and more abundant proteins (such as albumin) [7,17,27]. Subsequently, the supernatant (protein extract with proteins of molecular mass less than 90 kDa and with low albumin content) was separated by centrifugation, and ice-cold 80% (*v*/*v*) acetone solution (Merck, Rahway, NJ, USA) at a ratio of 1:4 (sample:acetone) was added to obtain protein pellets with molecular mass less than 90 kDa and with low albumin content [16,17]. The experimental procedures for liver proteome fractionation using 2D PAGE are briefly described as follows: first was the isoelectric focusing (IEF) step, in which the fractionation of proteins occurs by the isoelectric point (pI). Protein pellets obtained during the precipitation process were solubilized in denaturing and reducing solutions [7,16]. Three 13 cm IEF strips (pH gradient 3 to 10, Amersham Biosciences, Amersham, UK) were hydrated with 250 µL of protein extracts (obtained from the solubilization of protein pellets and diluted to contain 375 µg of protein) in their respective channels, in the hydration box and covered with mineral oil to prevent evaporation of the sample solution [7,16,17]. The hydration box was closed and maintained at room temperature for 12 h. After proper hydration, the strips were transferred to the Amersham Biosciences EPS 1001 isoelectric focusing system (Uppsala, Sweden) for the first-dimension electrophoretic run using the procedure described by Bataglioli et al. (2019) and Bittarello et al. (2020) [7,16]. The running time was 4.5 h. After the isoelectric focusing step, the IEF strips were rehydrated with two solutions. First, 12 mL of a solution containing 6 mol L^−1^ urea (Amersham Biosciences, Amersham, UK), 2% (*m*/*v*) sodium dodecyl sulfate (Amersham Biosciences, Amersham, UK), 30% (*v*/*v*) glycerol (Merck, Rahway, NJ, USA), 50 mmol L^−1^ Tris-HCl pH 8.8 (Amersham Biosciences, Amersham, UK) 0.002%, and bromophenol blue (Amersham Biosciences, Amersham, UK) was used. Additionally, 2% (*m*/*v*) *dithiothreitol (DTT,* J.T. Backer, Phillipsburg, NJ, USA) was used to maintain proteins in denatured and reduced forms. Subsequently, a solution similar to the previous one, but with DTT replaced by 2.50% (*m*/*v*) iodoacetamide (Amersham Biosciences, Amersham, UK), was used to promote the alkylation of the thiol groups and prevent the reoxidation of the proteins. The contact time between the two solutions and IEF strips under slight agitation on a shaking table was 15 min. Then, the second stage of the 2D PAGE was run, in which protein fractionation occurs as a function of the molecular mass; this was performed using the GE Healthcare Ettan™ DALT six 2D PAGE system (Uppsala, Sweden) and the experimental conditions described by Bittarello et al. 2020 and Santiago et al. 2023 [16,20]. At the end of the 2D PAGE runs, the proteins were fixed in gels using a solution containing 10% (*v*/*v*) acetic acid (J.T. Backer, Phillipsburg, NJ, USA) and 40% (*v*/*v*) ethanol, and the protein spots were visualized using colloidal Coomassie dye (Amersham Biosciences, Amersham, UK). After the protein spots were revealed, the gels were scanned, and the images were analyzed using the ImageMaster 2D Platinum 7.0 software and the procedures described by Santiago et al. 2023 [20].

### 3.4. Characterization of Hg/Se-Linked Protein Spots Using LC-MS/MS

Hg/Se-linked protein spots were extracted from the gels with the aid of a scalpel, cut into segments of approximately 1 mm^3^, transferred to microtubes containing 1 mL of 5% (*v*/*v*) acetic acid, and subjected to the following steps [31]: dye removal, protein reduction and alkylation, and tryptic digestion using 10 ng mL^−1^ trypsin solution. The extracts containing the peptides were transferred to new vials and concentrated to approximately 12 µL in Thermo Scientific equipment (Thermo Fisher Scientific, Waltham, MA, USA). The concentrated extracts were then passed through C18 ZipTip columns (Thermo Fisher Scientific, Waltham, MA, USA) for peptide cleanup and stored at −80 °C until further analysis for characterization using the nano ACQUITY UPLC-Xevo QTOF-MS system equipped with electrospray ionization (Waters, Manchester, UK), HSS T3 column (Acquity UPLC HSS T3 Waters, Manchester, UK) and operated in positive ion mode [18]. The data obtained from the mass spectra were processed and analyzed using the “Protein Lynx Global Server—PLGS software” (version 2.5) and by consulting the Uniprot database and the fish order Otophysi, to identify the proteins [7,16,17].

### 3.5. Analysis of Parameters Related to Oxidative Stress

To analyze the damage to cellular lipids and proteins resulting from oxidative stress, 0.20 g of liver tissue from each individual from the lower and higher Hg and Se concentration groups of *P. amazonica* and *R. vulpinus* (biological replicates) were subjected to the cell disruption process using Tris-HCl buffer pH 8.80 as extraction solution and the OMNI/analytic cell disruptor. Then, the extracts were centrifuged at 12,000× *g* for 30 min at 4 °C and the supernatants obtained were used to determine lipid lipoperoxidation (LPO) and glutathione peroxidase (GPx), catalase (CAT), and superoxide dismutase (SOD) activities using already consolidated methodologies from the literature [32,33,34,35].

### 3.6. Statistical Analysis

ImageMaster 2D Platinum software (version 7.0) (GE Healthcare) was used to analyze 2D PAGE gel images, as described by Santiago et al. 2023 [20]. Hg and Se determinations were expressed in M ± SD and subjected to Student’s *t* test and F test to identify any significant differences using SAS software (version 8) [7,17,20]. Regarding data from the analysis of CAT, SOD, and GPx activity and LPO concentrations, the Spearman Correlation test was used to evaluate the correlation of these parameters with C_Hgtotal_ and C_Setotal_ [16]. Proteomic data were analyzed using nanoAcquity UPLC Xevo QT of the MS Protein Lynx Global Server (PLGS) spectrometer software (version 2.5) [17].

## 4. Conclusions

The average molar ratio Hg:Se showed lower values in relation to the average molar ratio Se:Hg for the *P. amazonica* (non-predatory) and *R. vulpinus* (predatory) species, which reflected values of Se HBV > 5 for the two groups of the non-predatory species and >10 for the two groups of the predatory species, indicating that the organisms of both species had Se in their liver tissue to eliminate the toxic effects of Hg species. The metalloproteomic results indicated 11 protein bands in which enzymes and/or proteins associated with Hg and Se that play important functions in *P. amazonica* and *R. vulpinus* were identified. Moreover, the presented Se_total_ concentrations were approximately 50% higher than those of Hg_total_. Based on this, it can be inferred that, in *P. amazonica* and *R. vulpinus*, the formation of complexes could occur, forming inert *metal binding proteins* (*MBP*-R-[Hg-Se] and/or MPB-R2-[Hg-SE]); in this case, the binding of Se to Hg may mitigate the toxic effects of Hg species. Finally, the activities of SOD, CAT, and GPx, as well as LPO levels, were not significantly correlated with the Hg_total_ and Se_total_ concentrations, indicating, in principle, the non-occurrence of oxidative species induced by Hg species. This corroborates the metalloproteomics results. Thus, the insights reported in this study indicate that the consumption of *P. amazonica* and *R. vulpinus* as a source of protein for riverside populations in the Brazilian Amazon does not present a contamination risk by Hg species.

## Figures and Tables

**Table 1:** Hg_total_ and Se_total_ concentrations in the liver tissue samples from *P. amazonica*.

P. amazonica/NRC	Hg_total_ Concentration (µg kg^−1^)	Se_total_ Concentration (µg kg^−1^)
1	84.50 ± 2.500	126.6 ± 2.500
2	122.4 ± 3.040	149.3 ± 2.700
3	81.10 ± 0.9000	121.8 ± 2.200
4	123.5 ± 1.700	152.1 ± 2.800
5	120.9 ± 2.830	147.5 ± 2.600
6	86.70 ± 3.250	128.1 ± 2.300
7	87.80 ± 4.24	129.4 ± 2.400
8	125.5 ± 2.620	153.7 ± 2.800
DOLT-4	2.530 ± 0.04430 *	8.110 ± 0.1403 *

* Concentration—mg kg^−1^; DOLT-4 (Certified value—mg kg^−1^); C_Hg_ = 2.580 ± 0.2200; C_Se_ = 8.300 ± 1.300; Results expressed as a mean value ± standard deviation (*n* = 5, *p* < 0.05).

**Table 2:** Hg_total_ and Se_total_ concentrations in the liver tissue samples from *R. vulpinus*.

R. vulpinus/NRC	Hg_total_ Concentration (µg kg^−1^)	Se_total_ Concentration (µg kg^−1^)
1	143.2 ± 2.600	244.3 ± 4.400
2	586.6 ± 10.70	726.6 ± 12.80
3	136.4 ± 2.200	231.2 ± 3.900
4	146.5 ± 2.700	249.1 ± 4.600
5	555.6 ± 9.900	666.8 ± 11.30
6	645.9 ± 11.60	775.1 ± 13.20
7	156.8 ± 2.800	265.2 ± 4.700
8	528.3 ± 9.400	644.2 ± 10.90
DOLT-4	2.519 ± 0.04836 *	8.122 ± 0.1535 *

* Concentration—mg kg^−1^; DOLT-4 (Certified value—mg kg^−1^); C_Hg_ = 2.580 ± 0.2200; C_Se_ = 8.300 ± 1.300; Results expressed as a mean value ± standard deviation (*n* = 5, *p* < 0.05).

**Table 3:** Hg_total_ and Se_total_ concentrations in protein pellets from liver tissue of *P. amazonica* and *R. vulpinus*.

Groups Identification	Hgtotal Concentration (µg kg^−1^)	Setotal Concentration (µg kg^−1^)
*Pellet*-*Pa*-HgSe 1	81.44 ± 1.341	121.6 ± 2.100
*Pellet*-*Pa*-HgSe 2	118.2 ± 1.824	148.5 ± 2.314
*Pellet*-*Rv*-HgSe 1	137.8 ± 2.243	237.6 ± 3.971
*Pellet*-*Rv*-HgSe 2	564.6 ± 8.522	679.4 ± 9.342
DOLT-4	2.514 ± 0.04091 *	8.115 ± 0.1393 *

Pa-HgSe 1—Individual group of *P. amazonica* species with <[Hgtotal] and [Setotal]; Pa-HgSe 2—individuals group of *P. amazonica* species with >[Hgtotal] and [Setotal]; Rv-HgSe 1—individuals group of *R. vulpinus* species with <[Hgtotal] and [Setotal]; Rv-HgSe 2—g individual group of *R. vulpinus* group with >[Hgtotal] and [Setotal]; * Concentration—mg kg^−1^; DOLT-4 (Certified Value—mg kg^−1^); C_Hg_ = 2.580 ± 0.2200; C_Se_ = 8.300 ± 1.300. Results expressed as a mean value ± standard deviation (*n* = 5, *p* < 0.05).

**Table 4:** Mercury and selenium-associated proteins in protein spots of gels from liver tissues of *P. amazonica* and *R. vulpinus*.

ID. Spots	Protein Accession	Protein Description	Protein Score	C_Hg_ (mg kg^−1^)	C_Se_ (mg kg^−1^)
		***P. amazonica***			
**59**	P02140 P02018	Hemoglobin subunit beta Hemoglobin subunit alpha	2527.965 188.4514	9.700 ± 0.1400	16.15 ± 0.2100
**60**	P02017 P02140 P80856	Hemoglobin subunit alpha Hemoglobin subunit beta Fatty acid-binding protein_ liver	2305.963 1368.896 1131.812	17.24 ± 0.2300	33.41 ± 0.3900
**61**	P80856 Q9I8L5	Fatty acid-binding protein_ liver Fatty acid-binding protein 10-A_ liver basic	1522.318 1214.747	19.72 ± 0.2500	35.44 ± 0.4300
**69**	P83751	Actin_ cytoplasmic 1	3731.136	14.20 ± 0.1900	25.32 ± 0.3000
		***R. vulpinus***			
**1**	O73872	Superoxide dismutase [Cu-Zn]	615.6831	22.11 ± 0.3100	34.82 ± 0.4600
**4**	Q90XG0	Triosephosphate isomerase B	1373.102	24.20 ± 0.3600	39.20 ± 0.5100
**6**	Q90XG0	Triosephosphate isomerase B	4885.841	23.12 ± 0.3300	37.60 ± 0.4900
**8**	Q6NY77	4-hydroxy-2-oxoglutarate aldolase_ mitochondrial	126.5413	21.43 ± 0.2800	33.73 ± 0.4300
**10**	P83751 P49055	Actin_ cytoplasmic 1 Actin_ alpha skeletal muscle	8121.286 3804.837	28.44 ± 0.3100	43.12 ± 0.5100
**17**	Q9PTY0	ATP synthase subunit beta_ mitochondrial	128.7956	17.32 ± 0.2300	31.25 ± 0.4100
**26**	P18520 Q6NWF6	Intermediate filament protein ON3 Keratin_ type II cytoskeletal 8	144.8645 144.8645	14.35 ± 0.2100	26.82 ± 0.3100

C_Hg_, C_Se_—Results expressed as a mean value± standard deviation (*n* = 5, *p* < 0.05).

**Table 5:** Spearman correlation coefficients correlating Hg_total_ and Se_total_ concentrations with the activities of CAT, SOD, and GPx enzymes, and LPO concentration in liver tissue of *P. amazonica* and *R. vulpinus*.

Variables	LPO Sp.CC	CAT Sp.CC	SOD Sp.CC	GPx Sp.CC
*P. amazonica*	-	-	-	-
Group with <C_Hgtotal_	0.8425 *	−0.4597 ^NS^	0.6156 *	−0.8815 *
Group with >C_Hgtotal_	0.2697 ^NS^	0.1018 ^NS^	−0.7555 *	−0.7767 *
Group with <C_Setotal_	0.8427 *	−0.5095 ^NS^	0.6962 *	−0.8348 *
Group with >C_Setotal_	0.3634 ^NS^	0.2220 ^NS^	−0.6800 *	−0.8749 *
*R. vulpinus*	−	−	−	−
Group with <C_Hgtotal_	0.7342 *	−0.2536 ^NS^	0.2877 ^NS^	0.4822 ^NS^
Group with >C_Hgtotal_	0.3017 ^NS^	0.2864 ^NS^	−0.3387 ^NS^	−0.6000 *
Group with <C_Setotal_	0.7135 *	−0.3071 ^NS^	0.2328 ^NS^	0.4282 ^NS^
Group with >C_Setotal_	0.1301 ^NS^	0.2822 ^NS^	−0.4178 ^NS^	−0.6826 *

Sp.CC—Spearman correlation coefficients; * Significant (*p* < 0.05); ^NS^—Not significant (*p* ≥ 0.05).

## Data Availability

The datasets generated and analyzed during the current study are available in the Supplementary Materials.

## Supplementary Materials

The following supporting information can be downloaded at: https://www.mdpi.com/article/10.3390/ijms252211946/s1.
