# Supplementary material for: Metallomic Approach to Mercury and Selenium in the Liver Tissue of Psectrogaster amazonica and Raphiodon vulpinus from the Brazilian Amazon
Source: Int J Mol Sci. 2024 Nov 7;25(22):11946. doi: 10.3390/ijms252211946 (PMC11594490; doi:10.3390/ijms252211946)
Supplement: Supplementary file 1 [file ijms-25-11946-s001.zip › Supplementary Material Table S1.pdf]

Metallomic Approach to Mercury and Selenium in the Liver Tissue of *Psectrogaster amazonica* and *Raphiodon vulpinus* from the Brazilian Amazon

Izabela Bataglioli, José Vieira, Joyce da Siva, Luane Andrade, Victor Faria, Rebeca Corcoba, Ronaldo de Almeida, Luiz Zara, Marília Buzalaf, Jiri Adamec and Pedro Padilha

Table S1. Activity of SOD, CAT and GPx enzymes, and concentration of LPO determined in liver tissue of "individuals" of *P. amazonica* and *R. vulpinus* species.

Individuals	LPO - Concentration (nmol/g)	CAT - Activity (μmol/g)	SOD - Activity (nmol/g)	GPx - Activity (nmol/mg)
P. amazonica				
1	379.3±5.8	12.60±0.15	10.00±0.13	20.70±0.32
2	435.1±7.2	11.20±0.13	8.700±0.11	23.30±0.35
3	339.6±5.7	14.00±0.16	8.300±0.10	21.40±0.31
4	460.4±7.7	17.70±0.21	9.600±0.11	17.60±0.21
5	284.9±4.7	17.44±0.19	11.10±0.13	26.90±0.39
6	373.0±5.3	14.40±0.16	9.900±0.11	18.70±0.23
7	442.3±7.4	11.60±0.13	9.200±0.11	19.50±0.25
8	348.1±5.7	16.40±0.19	8.600±0.10	20.10±0.23
R. vulpinus				
1	468.8±7.9	2.700±0.03	2.800±0.04	8.800±0.11
2	332.9±5.6	3.400±0.04	3.000±0.04	7.300±0.11
3	444.1±7.8	5.200±0.06	3.500±0.06	9.600±0.14
4	402.6±6.6	4.200± 0.05	2.800±0.04	9.300±0.12
5	563.9±9.6	2.500±0.03	3.300±0.04	11.30±0.17
6	556.4±9.2	4.900±0.06	3.500±0.06	8.100±0.12
7	583.8±9.8	4.100±0.05	3.700±0.05	10.00±0.15
8	419.1±7.5	4.800±0.06	3.900±0.07	9.400±0.16
